# Clinical and genomic features of a Listeria monocytogenes fatal case of meningitis in Madagascar

**DOI:** 10.1099/acmi.0.000764.v3

**Published:** 2024-06-27

**Authors:** Saïda Rasoanandrasana, Mamitina Alain Noah Rabenandrasana, Lucia Mélanie Ravaoharisoa, Narindra Randrianaivo, Vonintsoa Lalaina Rahajamanana, Zafindrasoa Domoina Rakotovao-Ravahatra, Alexandra Moura, Marc Lecuit, Andriamiadana Luc Rakotovao

**Affiliations:** 1 Bacteriology Laboratory, CHU Joseph Raseta Befelatanana, Antananarivo, Madagascar; 2 Experimental Bacteriology Unit, Institut Pasteur of Madagascar, Antananarivo, Madagascar; 3 Pediatric Service, CHU Joseph Raseta Befelatanana, Antananarivo, Madagascar; 4 Bacteriology Laboratory, Centre Hospitalier Universitaire Mère Enfant Tsaralàlana, Antananarivo, Madagascar; 5 Institut Pasteur, Biology of Infection Unit, Université Paris Cité, Inserm U1117, Paris, 75015, France; 6 Institut Pasteur, National Reference Centre and WHO Collaborating Centre Listeria, Paris, France; 7 Division of Infectious Diseases and Tropical Medicine, Institut Imagine, APHP, Necker-Enfants Malades University Hospital, Paris, France

**Keywords:** fatal case, genomic characterization, listeriosis, Madagascar, meningitis

## Abstract

Listeriosis constitutes a significant public health threat due to its high mortality rate. This study investigates the microbiological and genomic characteristics of *Listeria monocytogenes* isolates in Madagascar, where listeriosis is a notifiable disease. The analysis focuses on a fatal case of meningeal listeriosis in a 12-year-old child. Genomic analysis revealed a novel cgMLST type (L2-SL8-ST8-CT11697; CC8, serogroup Iia) with typical virulence and antibiotic resistance profiles. These isolates, unique to Madagascar, formed an independent clade in the phylogenetic tree. This study presents the first genomic characterization of Listeria isolates in Madagascar, highlighting the necessity of ongoing genomic surveillance to strengthen listeriosis prevention and control strategies in the region.

**Impact Statement**

This study’s significant findings on *Listeria monocytogenes* infections in Madagascar highlight the elevated risk among specific populations, particularly pregnant women from sub-Saharan Africa and the Maghreb region. The research reveals previously undocumented manifestations of the disease, expanding our understanding of its clinical presentation. Crucially, genomic analysis has identified a novel strain unique to Madagascar, providing valuable insights for tracing the pathogen’s origins and transmission. The presence of specific virulence genes and antibiotic resistance profiles underscores the need for tailored prevention strategies. This pioneering genomic characterization lays the groundwork for targeted interventions and enhances public health initiatives, ultimately contributing to the prevention of listeriosis in the region.

## Data Summary

The assemblies and SRA data are deposited on NCBI under the bioproject PRJNA1032442 (all accession numbers are listed in the supplementary table, avaliable with the online version of this article).

## Introduction

Listeriosis is one of the most serious food-borne illnesses, caused by the Gram-positive bacteria *Listeria monocytogenes*. It is a relatively rare disease, with 0.1 to 10 cases per million inhabitants per year, depending on the country and region. Although the number of listeriosis cases is low, the high mortality rate associated with this infection makes it a worrying public health issue [1]. In pregnant women, listeriosis is higher in populations from sub-Saharan Africa and the Maghreb than in those from Latin America and the Caribbean. Among the 107 cases of maternal-neonatal listeriosis, 35 (33 %) originated from the Maghreb or sub-Saharan Africa, a rate three times higher than that observed in the general pregnant population in France in 2010, as indicated by national records (88 000 [11 %] out of 832 000 women, *P*<0.0001) [2].

Listeriosis is a notifiable disease in Madagascar. A recent study [3] documented the case of a 75-year-old man with an aortic bioprosthesis, who was admitted with polyarthritis in an afebrile state. Blood cultures confirmed the presence of *L. monocytogenes*, leading to the diagnosis of *Listeria* endocarditis and spondylodiscitis, both uncommon manifestations of listeriosis. The patient showed improvement following antibiotic therapy [3]. Here we describe a case of invasive listeriosis at the CHU Befelatanana in Madagascar.

## Case presentation

**Fig. 1.**
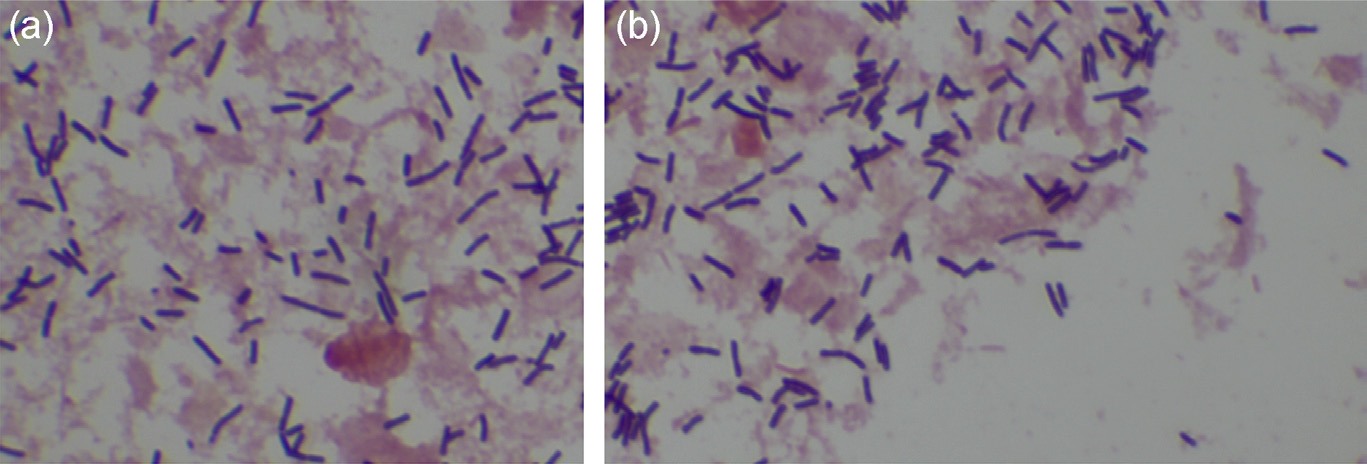
Gram-staining of *Listeria monocytogenes* strains (**a**) isolated from cerebrospinal fluid (CSF) and blood cultures (**b**).

A 12-year-old patient was admitted for meningeal syndrome, with a history of hyperthermic convulsive seizure at age 3. The patient is the first child in a sibling group of three, with an up-to-date immunization schedule. There was no similar case in the family. We received a sample of the cerebrospinal fluid and blood cultures. Direct examination of the CSF revealed hyperleukocytosis with a predominance of polynuclear neutrophils, hyperproteinorrachia, and hypoglycorrachia. Solid and liquid cultures were positive for a Gram-positive bacillus, which was identified as *L. monocytogenes* using the BioFire FilmArray Meningitis/Encephalitis (ME) Panel (Biomérieux, Marcy-l'Étoile, France). Concurrently, the blood culture also grew a Gram-positive bacillus with a 48 h growth period. Unfortunately, despite appropriate treatment according to the Common Infections Guidelines in Madagascar, 2019 edition [4] with ceftriaxone, gentamicin, and vancomycin, the patient did not survive. The antimicrobial susceptibility testing (AST) is not performed at the time of patient admission due to cost constraints for patients, especially considering the urgency of promptly treating bacterial meningitis, particularly in children. We used methods such as Gram-staining and Matrix-assisted laser desorption/ionization – time of flight mass spectrometry (MALDI-TOF MS) using the MALDI-TOF Biotyper System with the BDAL spectra database containing 8 468 MSPs, and the IVD containing 8 326 MSPs, for bacterial identification and the extraction method with 70 % formic acid and acetonitrile. The results indicated the presence of Gram-positive bacteria (Fig. 1), with the identification as *L. monocytogenes* with a score of >2.0 (Table 1). We notified the appropriate authorities; however, as of our current understanding, the origin remains undetermined.

**Table 1.** Identification results on the MALDI-TOF MS for the two strains of *Listeria monocytogenes*. If the score is between: 2.00–3.00, the identification is performed with a high confidence level; 1.70–1.99, the identification is performed with a low confidence level; 0.00–1.69, no identification is attributed to the organism

Strains origins	Corresponding reference mass spectra	Score	NCBI identifier
Cerebrospinal fluid	*Listeria monocytogenes* CCUG 32843 CCUG	2.14	1639
	*Listeria monocytogenes* CCUG 59664 CCUG	2.14	1639
	*Listeria monocytogenes* CCUG 31527 CCUG	2.09	1639
	*Listeria monocytogenes* CCUG 35751 CCUG	2.08	1639
	*Listeria monocytogenes* DSM 20600T DSM_1	2.07	1639
	*Listeria monocytogenes* CCUG 61052 CCUG	2.07	1639
	*Listeria monocytogenes* Mb19348_1 CHB_1	2.06	1639
	*Listeria monocytogenes* CCUG 49753 CCUG	2.03	1639
	*Listeria monocytogenes* ser4B ATCC 19115 THL_1	2.02	1639
	*Listeria monocytogenes* CCUG 32964A CCUG	2.02	1639
Blood culture	*Listeria monocytogenes* CCUG 59664 CCUG	2.21	1639
	*Listeria monocytogenes* CCUG 32843 CCUG	2.12	1639
	*Listeria monocytogenes* CCUG 49753 CCUG	2.12	1639
	*Listeria monocytogenes* CCUG 35751 CCUG	2.11	1639
	*Listeria monocytogenes* CCUG 31527 CCUG	2.1	1639
	*Listeria monocytogenes* CCUG 61052 CCUG	2.1	1639
	*Listeria monocytogenes* CCUG 33548 CCUG	2.09	1639
	*Listeria monocytogenes* CCUG 32964A CCUG	2.09	1639
	*Listeria monocytogenes* Mb19348_1 CHB_1	2.08	1639
	*Listeria monocytogenes* CCUG 61052 CCUG	2.03	1639

Isolate characterization was performed by whole genome sequencing with Illumina technology and genomic analysis with TORMES pipeline [5] and VirulenceFinder [6,7]. PCR-serogrouping, multilocus sequencing typing (MLST), core genome MLST (cgMLST) and virulence and resistance profiles were performed using BIGSdb-*Listeria* [6,8,14]. We conducted a phylogenetic analysis using core genome alignments that were not purged for genetic recombination, obtained with Parsnp [15], incorporating publicly available strains from the BIGSdb-*Listeria* database belonging to clonal complex CC8 (as of 9 September 2023).

The two isolates of *L. monocytogenes* isolates belonged to the same cgMLST type (L2-SL8-ST8-CT11697; CC8, serogroup Iia), which is new in BIGSdb-*Listeria* database. The typical *L. monocytogenes* virulence genes were present and included the pathogenic island LIPI-1 [16], as well the *inlAB* operon necessary for gut and brain invasion [16]. Intrinsic antibiotic resistance (*fosX, lin, norB, sul, pbp*) genes were present, and no acquired resistance was detected [16]. Both isolates carried the stress island SSI-1 [16], which favours adaptation to low pH and high salt concentration [16] and LGI-3 [16].

**Fig. 2.**
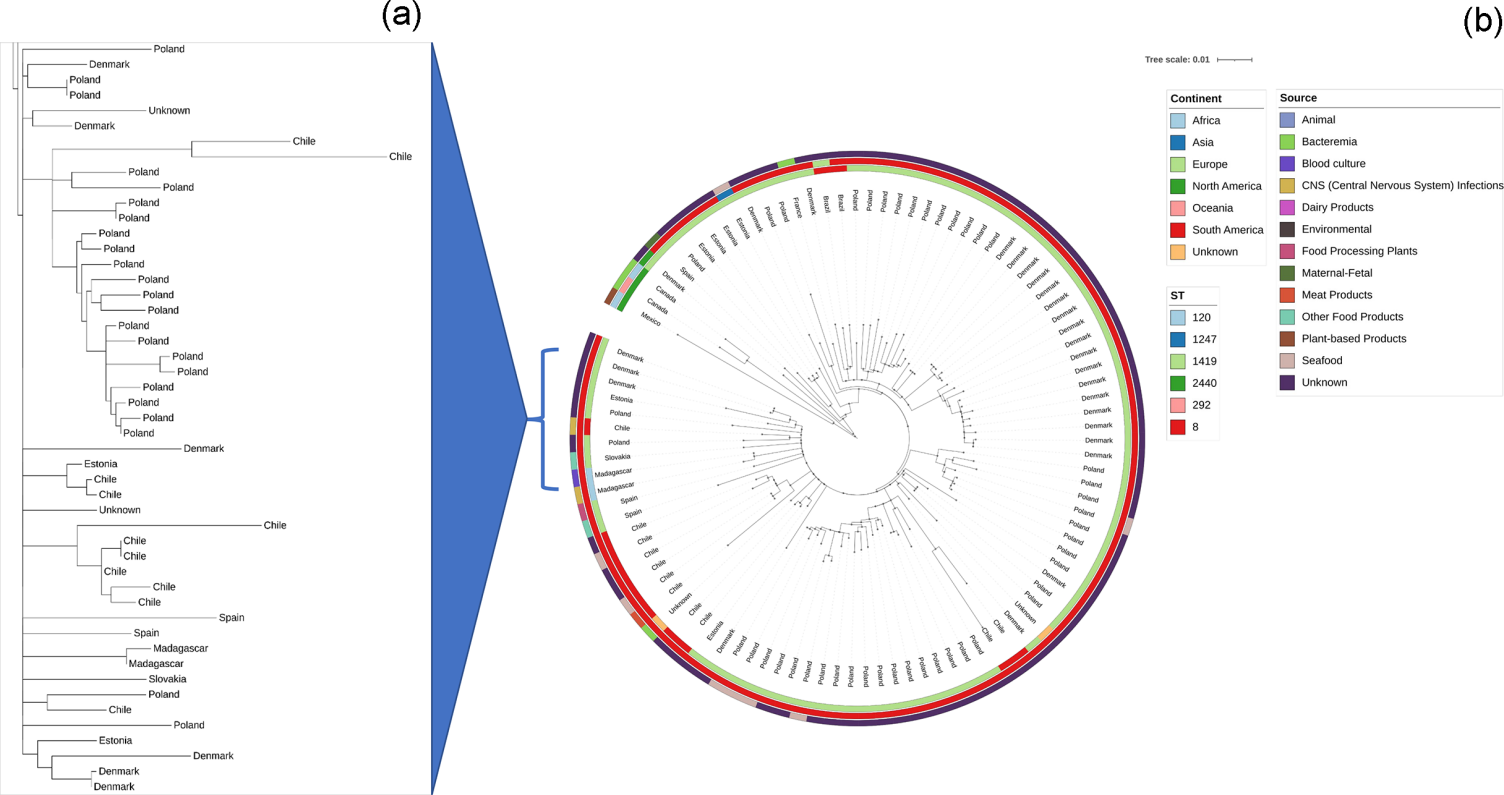
Phylogenetic tree based on the core genome alignment of Malagasy isolates and 100 publicly available CC8 sublineage SL8 genomes from BIGSdb-Listeria (as of 9 September 2023). From the innermost to the outermost circle, the first circle represents the distribution by ST, the second represents the distribution by continent, and the last one represents the isolation sources (**b**) with a zoom on the clades containing strains from Madagascar (**a**).

This new type differs by 12 allelic differences from L2-SL8-ST8-CT750, which has been found in Europe (Denmark, Poland, Estonia) and Latin America (Chile). Our phylogenetic analysis (Fig. 2) confirms that the Malagasy isolates exhibit a high degree of genetic similarity and form an independent clade, distinct from those originating in other countries.

## Discussion

To the best of our knowledge, this work represents the first genomic characterization of *Listeria* isolates from Madagascar. Further sequencing efforts will help to identify sources of contamination and improve listeriosis surveillance and prevention efforts in our region. The discovery of a novel strain specific to Madagascar highlights the uniqueness of *Listeria* circulating in the local population. This groundbreaking research sets the stage for further sequencing efforts, aiming to identify contamination sources and enhance listeriosis surveillance and prevention strategies within Madagascar. The findings underscore the importance of integrating genomic data into public health initiatives, enabling targeted interventions, swift outbreak responses, and tailored patient care. Ultimately, this study serves as a crucial foundation for comprehensive research and collaborative efforts to mitigate the impact of listeriosis in Madagascar.

## supplementary material

10.1099/acmi.0.000764.v3 Supplementary Material.

## Abbreviations

BDAL: Bruker Daltonics Library
BIGSdb: bacterial isolate genome sequence database
cgMLST: core genome multi-locus sequence typing
CSF: cerebrospinal fluid
IVD: in vitro diagnostic
MALDI-TOF MS: matrix-assisted laser desorption/ionization time-of-flight mass spectrometry
MLST: multi-locus sequence typing
MSP: main spectral profiles
PCR: polymerase chain reaction
